# Identification of *Kosakonia cowanii* as a rare cause of acute cholecystitis: case report and review of the literature

**DOI:** 10.1186/s12879-020-05084-6

**Published:** 2020-05-24

**Authors:** Benjamin Berinson, Eugen Bellon, Martin Christner, Anna Both, Martin Aepfelbacher, Holger Rohde

**Affiliations:** 1 Institute for Medical Microbiology, Virology and Hygiene, University Medical Centre Hamburg-Eppendorf, Hamburg, Germany; 2 Department for Visceral-Surgery, University Medical Centre Hamburg-Eppendorf, Hamburg, Germany

**Keywords:** Cholecystitis, Diagnostics, Species identification, *Kosakonia cowanii*, Whole genome sequencing, Case report

## Abstract

**Background:**

*Kosakonia cowanii*, formerly known as *Enterobacter cowanii*, is a Gram-negative bacillus belonging to the order *Enterobacterales*. The species is usually recognized as a plant pathogen and has only anecdotally been encountered as a human pathogen. Here we describe the rare case of a *K. cowanii* infection presenting as an acute cholecystitis and provide a review of available literature. Evident difficulties in species identification by biochemical profiling suggests that potentially, *K. cowanii* might represent an underestimated human pathogen.

**Case presentation:**

A 61-year old immunocompromised man presented to the hospital with fever and pain in the upper right abdomen. Sonography revealed an inflamed gall bladder and several gall stones. A cholecystectomy proved diagnosis of an acute cholecystitis with a partial necrosis of the gall bladder. Surgical specimen grew pure cultures of Gram-negative rods unambiguously identified as *K. cowanii* by MALDI-TOF, 16S-rRNA analysis and whole genome sequencing.

**Conclusions:**

Reporting cases of *Kosakonia* species can shed light on the prevalence and clinical importance of this rare cause of human infection. Our case is the first to describe an infection without prior traumatic inoculation of the pathogen from its usual habitat, a plant, to the patient. This raises the question of the route of infections as well as the pathogen’s ability to colonize the human gut.

## Background

Formerly, isolates of the species, now referred to as *Kosakonia cowanii*, had been placed within the species of *Enterobacter agglomerans/Pantoea agglomerans*. In the year 2000, the isolates were reclassified into the newly designated species of *Enterobacter cowanii* [1]. Brady et al. finally proposed the reclassification of the isolates under the name of *Kosakonia cowanii* based on multi-locus sequence analysis (Brady et al., 2013). *K. cowanii* is a well-recognized plant pathogen, causing a variety of infections resulting in e.g. wilt and dieback [2]. Although the primary description of *K. cowanii* as a distinct species also was based on isolates obtained from human specimens, the pathogenic potential in humans remains elusive. Only recently, *K. cowanii* has been described as a causative agent of rhabdomyolysis and bacteremia related to a rose thorn prick, indicating that infections from exogenous sources can occur [3]. In this report, *K. cowanii* was identified in two independent intraoperative samples obtained from an immunocompromised patient undergoing cholecystectomy for acute cholecystitis.

## Case presentation

A 61-year-old man presented to the emergency department with recurrent fever of up to 38.9 °C since 4 days, nausea and pain in the upper right abdomen. He had a history of type 2 diabetes mellitus, adequately controlled with Metformin, hypertension and mild osteoporosis. Furthermore, he suffered from psoriatic arthritis, treated with Golimumab 50 mg once per month, Methotrexate 15 mg once per week and Prednisolone 7.5 mg daily.

Physical examination revealed a tender right upper quadrant and positive Murphy’s sign, while vital signs were normal. Sonography of the abdomen found several gallstones, sludge and thickening of the gallbladder wall of 7 mm. Leucocyte count was 11.9 × 10^9^ /L and C reactive protein 144 mg/dl. Alkaline phosphatase was elevated at 241 U/l, gamma-glutamyl-transferase at 285 U/l and bilirubin at 2.1 mg/dl. According to local guidelines for the treatment of acute cholecystitis, antimicrobial therapy was initiated with intravenous cefuroxime and metronidazole, and a cholecystectomy was performed. Intraoperatively, the diagnosis of acute cholecystitis was confirmed. Partial necrosis of the gallbladder wall and purulent fluid in the right upper abdomen were evident. Cholecystectomy and extensive irrigation of the abdomen were performed laparoscopically without complications. Histology of the removed gallbladder revealed an ulcerophlegmonous cholecystitis with necrotic parts.

The patient became afebrile one day after surgery. The post-operative course was uncomplicated and the patient was discharged after 3 days. Antimicrobial therapy was changed to an oral application route and continued for additional seven days.

## Microbiology

Two independent samples were collected during surgery, one swab from the inside of the gallbladder and one bile sample. Specimens were sent for routine microbiology diagnostics and streaked onto Columbia blood agar and MacConkey agar for aerobic and Schaedler agar for anaerobic incubation, respectively (all media Oxoid, Basingstoke, UK). After 24 h of aerobic and anaerobic incubation at 37 °C, both samples grew pure cultures of a Gram-negative bacillus, identified by matrix-assisted laser desorption ionization/time off light mass spectrometry fingerprinting (MALDI-TOF; Bruker, Bremen, Germany) as *K. cowanii*.

Species identification was confirmed by partial sequencing of the 16S ribosomal RNA (rRNA) gene, which showed 100% identity of non-ambiguous bases to the 16S rRNA of *K. cowanii type* strain 888–76 (GenBank accession number CP019445.1) [4]. Notably, biochemical species-level identification, performed with VITEK2 GN ID card (Biomerieux, Marcy-l’Etoile, France), incorrectly identified the strain as *Pantoea spp*. with a certainty of 98%.

Minimal inhibitory concentrations (MICs) were determined by gradient diffusion (Etest, Biomerieux, Marcy-l’Etoile, France; Liofilchem, Roseto degli Abruzzi, Italy), and verified by broth microdilution (Micronaut-S MDR MRGN-Screening 2 and Micronaut-S β-Lactamases, Merlin Diagnostika, Bornheim, Germany) (Table 1).

**Table 1:** Antimicrobial susceptibility profile of *K. cowanii* from a cholecystitis

Antibiotic substance	MIC^a^ [mg/mL]	Interpretation^b^
Ampicillin	≥ 256	R
Ampicillin/Sulbactam	≤ 2	S
Cefoxitin	4	Wildtype
Piperacillin	8	S
Piperacillin/Tazobactam	≤ 0,5	S
Fosfomycin	≥ 64	R
Meropenem	≤ 0.0032	S
Ciprofloxacin	≤ 0.04	S
Colistin	≤ 1	S

^a^MICs as determined by Etest and broth microdilution for *K. cowanii* clinical isolate

^b^Interpretation according to EUCAST criteria for *Enterobacterales* (The European Committee on Antimicrobial Susceptibility Testing. Breakpoint tables for interpretation of MICs and zone diameters. Version 9.0, 2018. http://eucast.org)

Whole genome sequencing of the isolate was performed using the Illumina NextSeq platform. Reads were assembled with spades [5] and annotated with prokka [6]. The draft genome comprises 50 contigs larger than 200 base pairs with a total length of 4.9 million base pairs and a G + C-content of 56.2%. Bioinformatic analysis showed 98.4% average nucleotide identity to *K. cowanii* 888–76 [7], and a genome to genome distance between the two strains corresponding to a DNA-DNA hybridization relative binding ratio of 86.7% [8], thus confirming MALDI-TOF- and 16S-based species-level identification. The virulence gene profile determined with abricate (https://github.com/tseemann/abricate) and the virulence factor database [9] matched the profile of the *K. cowanii* type strain 887–76, which also originates from a clinical sample. In agreement with phenotypic testing (Table 1) resistance gene profiling identified a class A beta-lactamase showing 98.6% amino acid identity to the beta-lactamase of *K. cowanii* 888–76 (Genebank accession number APZ06536.1) but no class C beta-lactamase as typically encountered in other Enterobacter-like species. Sequencing reads have been deposited in NCBI’s small reads archive (SRR8572161). The strain was deposited at the German Collection of Microorganisms and Cell Cultures GmbH (DSMZ) (reference number DSM 108916).

## Discussion and conclusions

Acute cholecystitis, which is a sudden inflammation of the gallbladder, is a common diagnosis worldwide. This disease is in more than 90% of the cases a complication of a cholecystolithiasis, complicated by an infection with predominantly Gram-negative bacteria of the order of *Enterobacterales* [10]. In Germany more than 63.000 cases of cholecystitis were reported in 2010 [11]. Cholecystitis is regarded as a typical endogenous infection caused by pathogens derived from the patient’s microbiota.

Here we describe the first case of cholecystitis caused by *K. cowanii* in an immunocompromised man. Conventionally, *K. cowanii* is regarded as a plant pathogen and probably only rarely causes human infections. In fact, to our knowledge only one case report so far unambiguously identified *K. cowanii* as a causative agent in the context of a human infection. In that patient, rhabdomyolysis and bacteremia occurred after a rose thorn prick, i.e. most likely resulted from an exogenous source potentially contaminated with *K. cowanii* [3]. In contrast, in our case *K. cowanii* caused an endogenous infection. This could have potentially resulted from ingestion of food containing *K. cowanii*, resulting in a transient colonization of the patient’s gut. Studies in the past have shown, that *K. cowanii* is a food contaminant, shown by its presence in powdered infant formula milk and on edible flowers or basil leaves [12, 13]. Further studies are needed to clarify if *K. cowanii* also might be able to permanently colonize the human intestine.

As so far described, patients receiving a powerful immunomodulatory medication of Golimumab and Methotrexate develop more infections than a placebo controlled group. Higher rates of infections also occurred with higher doses of Golimumab. These infections usually consist of respiratory infections up to pneumonia or urinary tract, but cases of acute cholecystitis also have been described [14, 15]. One can therefore argue that the administered medication might have facilitated the development of the infection with this rather uncommon pathogen.

It needs to be stressed that the species most probably will be misidentified if conventional biochemical differentiation systems are being employed, potentially leading to an underestimation of *K. cowanii*’s true importance as a human pathogen. Intriguingly, a retrospective study identified 53 pediatric cases of infection with *Pantoea agglomerans*, of which 7 had a penetrating trauma with a plant thorn, stick or glass shard [16]. A more thorough analysis of infections in which *Pantoea agglomerans* is tentatively identified as the causative agent might in fact reveal *K. cowanii* as the real pathogen. As already observed with other pathogens, the broad implementation of MALDI-TOF whole cell mass fingerprinting technology as the first line technique for routine microbiological differentiation might also shed light on the prevalence of *K. cowanii* and other plant pathogens in human infections.

## Data Availability

The isolate was submitted to the German Collection of Microorganisms and Cell Cultures (DSMZ) as DSM 108916. The isolate’s 16S sequence was deposited to NCBI GenBank under accession number MK517531. Next generation sequencing data was deposited to NCBI’s sequence read archive (SRA) under BioProject number PRJNA522304.

## Abbreviations

MALDI-TOF: Matrix-assisted laser desorption/ionization - time of flight
DSMZ: German Collection of Microorganisms and Cell Cultures GmbH
MIC: minimal inhibitory concentration
rRNA: ribosomal ribonucleic acid
DNA: Deoxyribonucleic acid
EUCAST: European Committee on Antimicrobial Susceptibility Testing
